# Retrospective analysis of image characteristics of 76 cases of cerebral vascular fenestrations

**DOI:** 10.3389/fneur.2022.986167

**Published:** 2022-12-08

**Authors:** Luyao Xu, Xiaodi Chen, Wei Xiang, Hongchun Wei, Zhigang Liang

**Affiliations:** Department of Neurology, Yantai Yuhuangding Hospital, Qingdao University, Yantai, China

**Keywords:** cerebral vascular fenestration, aneurysm, cerebral infarction, clinical relevance, image characteristics

## Abstract

**Objectives:**

This study aims to summarize the clinical and imaging features of cerebral vascular fenestration and to explore the association between cerebral vascular fenestration and cerebral infarction and aneurysm.

**Materials and methods:**

The computed tomography angiography (CTA), magnetic resonance angiography (MRA), and digital subtraction angiography (DSA) imaging data of 76 cases of cerebral vascular fenestration from January 2021 and December 2021 in the Yantai Yuhuangding Hospital Affiliated to Qingdao University were analyzed. The general information was described. The location, morphology, and size of cerebral vascular fenestration were described. The association between cerebral vascular fenestration and infarction and aneurysm was analyzed.

**Results:**

Among 76 patients, a total of 80 fenestrations were detected (two patients had three fenestrations), and basilar artery fenestration was the most common (28/80). The fenestration <5 mm was 43/80, 5–10 mm was 12/80, and ≥10 mm was 25/80. Moreover, 19 patients had other vascular diseases: 10 with aneurysms, 5 with moyamoya diseases, and 4 with cerebral artery dissections. Except for one aneurysm at the site of the fenestration, other aneurysms were separate from the fenestrations. In addition, 37 patients had cerebral infarctions, of which 16 had cerebral infarctions in the blood supply area of the arterial fenestration. Among these 16 patients, there was more cerebral infarction in posterior circulation than in anterior circulation. However, no statistically significant differences were detected in the risk factors between the fenestration-relevant cerebral infarctions group and the non-fenestration-relevant cerebral infarctions group.

**Conclusion:**

In our study, cerebral vascular fenestration occurred most frequently in the basilar artery and may be combined with other vascular diseases. Fenestration in posterior circulation may be related to cerebrovascular diseases. Nonetheless, no clear clinical relevance was observed between fenestration and cerebral infarction. Also, we did not find a definite association between fenestration and aneurysm. For fenestration patients with cerebral infarctions, long-term antiplatelet and statin therapy may be safe and effective.

## Introduction

Fenestration of the intracranial artery is segmental duplications of the lumen into two distinct channels, each comprising endothelial and muscular layers with or without a shared adventitia (1). Fenestrations result from partial failure of fusion of paired primitive embryologic vessels or incomplete obliteration of anastomosis in a primitive vascular network (2). Varying series from autopsy, surgical dissection, DSA, CTA, and MRA imaging have suggested a widely varying prevalence of fenestrations ranging from 0.7 to 60% (3–6). It is difficult to define the true incidence of cerebral vascular fenestration as it greatly depends on the type and methods of fenestration detection. According to the length of the fenestrated vessel branch, fenestration is divided into three types, namely, length <5, 5 mm ≤ length ≤ 10 mm, and length ≥10 mm. Based on the morphology, fenestration is divided into four variants, namely, slit-like shape, convex lens-like shape, duplicated artery, and other irregular shape fenestrations (7). Each type of fenestration has specific characteristics. Slit-like fenestrations are small with inconspicuous openings, whereas convex lens-like fenestrations present long and wide openings (3). The duplication variant refers to the total duplication of an artery (8). The irregularly-shaped fenestrations cannot be included in the first three categories.

The fenestrations of the cerebral arteries appear in all the branches but are most commonly reported in the vertebrobasilar artery (4), ACA (9), MCA (10–12), ACOA (13), and the posterior cerebral artery (14). In recent years, more and more people were found to suffer from fenestration, as we all know, the vessels where the fenestration is located will have hemodynamic changes, and whether this will cause the occurrence of other diseases is still unknown. Among the reported cases, patients with fenestrations were often accompanied by other vascular diseases, such as intracerebral aneurysm, moyamoya disease, intracranial arteriovenous malformation, and cerebral ischemia. Many people with fenestration are extremely worried about this. However, whether the occurrence of these diseases is related to fenestration is yet to be clarified. Therefore, in this study, we aimed to explore the clinical relevance between cerebral vascular fenestration and other associated cerebral vascular diseases.

## Materials and methods

### Study population

The study was performed according to the guidelines from the Helsinki Declaration and was approved by the Institutional Review Board of the Hospital. All patients or their legally authorized guardians provided informed consent according to the requirements of the Ethics Committee. Herein, patients whose image findings were compatible with cerebral vascular fenestration in our hospital from January–December 2021 were analyzed in this retrospective study.

### Baseline radiological characteristics

All the patients underwent DSA or MRA or CTA in our hospital to confirm the diagnosis of cerebral vascular fenestration. The location, morphology, and size of cerebral vascular fenestration were described. Based on the length of the fenestrated vessel branch, fenestration is divided into three types and based on the morphology, fenestration is divided into four variants. According to the correlation between BA and the location of the origin of the anterior inferior cerebellar artery (AICA), BA fenestrations were divided into four types, namely, type I, the fenestration is located proximal to the AICA or absence of AICA; type II, bilateral AICAs symmetrically originating from the fenestrated trunks; type III, a unilateral AICA originating from one side of the fenestrated trunk; and type IV, the fenestration is located distal to the AICA (8). All 37 patients with cerebral infarctions underwent magnetic resonance imaging, which confirmed the underlying cause. The imaging data were carefully analyzed by two neurologists and one experienced radiologist. The discrepancies in whether a fenestration existed or the morphology were resolved by discussion or acquiring an external opinion with other experienced neurologists.

### Treatment and clinical outcomes

The 37 cerebral vascular fenestration patients with cerebral infarction received the dual antiplatelet and lipid regulation treatment during hospitalization (about 7 days). This treatment was continued 3 months after discharge (the specific medication time shall be adjusted according to patients' conditions). A routine follow-up was conducted for 1 year through outpatient monitoring or direct telephonic contact with the patients or their relatives.

### Statistical analysis

All statistical analyses were performed using a commercial statistical software package (SPSS for Windows, version 26.0, IBM-SPSS, Chicago, IL, USA). Continuous variables were expressed as mean ± standard deviation (SD). Mann–Whitney U-test was used to analyze the difference in age between the fenestration-relevant cerebral infarctions group and the non-fenestration-relevant cerebral infarctions group. Fisher's precision probability test was performed in between-group comparisons of risk factors, including sex, history of stroke, hypertension, diabetes mellitus, dyslipidemia, atrial fibrillation, cerebral artery stenosis, hyperhomocysteinemia, coronary atherosclerotic heart disease, and recurrence of cerebral infarction. Differences with *P* < 0.05 were deemed statistically significant.

## Results

### Patient baseline characteristics

From January 2021 to December 2021, 76 patients with cerebral vascular fenestration were included, including 44 (57.9%) men and 32 (42.1%) women. The age of the cohort ranged from 24–79 (53.9 ± 28.6) years old. Among these patients, 37 had cerebral infarctions, 10 had aneurysms, 5 presented moyamoya disease, and 4 had cerebral artery dissection. All baseline characteristics are shown in Table 1. The demographic information, including patients' sex, age, infarction site, cerebral vascular stenosis, the location of fenestrations, and the etiology of stroke [according to Trial of Org 10172 in Acute Stroke Treatment (TOAST) criteria] of fenestration patients with cerebral infarction was collected and analyzed (Table 2). We analyzed the difference in age in fenestration located in the anterior or posterior circulation (Table 3).

**Table 1:** Baseline characteristics of 76 patients with cerebral artery fenestrations.

**Variable**	**Value**
Male, *n* (%)	44 (57.9%)
Age, mean ± SD	53.9 ± 28.6
**Risk factors**	
Hypertension, *n* (%)	44 (57.9%)
Diabetes mellitus, *n* (%)	23 (30.3%)
Dyslipidemia, *n* (%)	8 (10.5%)
Atrial fibrillation	2 (2.6%)
Smoking, *n* (%)	34 (44.7%)
**Radiographic abnormality**	
Cerebral ischemia	37 (48.7%)
Cerebral aneurysm	10 (13.2%)
Moyamoya disease	5 (6.6%)
Cerebral artery dissection	4 (5.3%)
Cerebral artery stenosis	49 (64.5%)

**Table 2:** Demographic and clinical characteristics of patients with fenestration-relevant cerebral infarctions.

**Case**	**Age, y**	**Sex**	**Site of fenestration**	**Infarction location**	**Risk factors**	**Cerebral artery stenosis**	**TOAST**
1	54	M	BA	Cerebellum	HTN, dyslipidemia, DM	BA	LAA
2	44	M	Left, VA	Cerebellum	HTN, DM	Bilateral MCA	LAA
3	71	M	BA	Cerebellum	DM	BA	LAA
4	59	M	Right, VA; BA	Dorsal thalamus	NA	Right, VA; BA	LAA
5	75	F	Right, VA	Pons	HTN, DM	Left, VA; Right, ICA	LAA
6	66	M	Right, VA	Thalamus	HTN	BA	LAA
7	69	F	Left, MCA	Left corona radiata	HTN, DM	Left, CCA	LAA
8	47	M	Left, MCA	Left frontal lobe, parietal lobe, temporal lobe and occipital lobe	Dyslipidemia	Right, ACA	LAA
9	74	M	Left, VA	Cerebellum	HTN, DM	Left, VA; Left, ICA; Right, MCA	LAA
10	68	M	BA	Pons	HTN	BA; Right, VA	LAA
11	64	f	BA	Cerebellum, medulla oblongata	HTN	Right, MCA; Right, ICA; Left, ICA	LAA
12	51	M	BA	Pons, thalamus	HTN	Right, ICA;	SUE
13	63	F	BA	Cerebellum, pons	NA	BA; Right, ACA	LAA
14	79	M	Right, VA	Cerebellum	HTN, DM	Right, ICA; Left, VA; Left, MCA	LAA
15	46	F	Right, VA	Cerebellum, pons	HTN	NA	LAA
16	41	M	Left, VA	Brainstem	NA	NA	LAA

F, female; M, male; HTN, hypertension; DM, diabetes mellitus; NA, not applicable; ACA, anterior cerebral artery; MCA, middle cerebral artery; ACOA, anterior communicating artery; VA, vertebral artery; BA, basilar artery; ICA, internal carotid artery; PICA, posterior inferior cerebellar artery; LAA, large-artery atherosclerosis; TOAST, Trial of Org 10,172 Acute Stroke Treatment; SUE, stroke of undetermined etiology.

**Table 3:** Differences in the age in fenestration located in the anterior and posterior circulations.

**Age/location**	**<60 years**	**>60 years**
Anterior circulation	13 (54.2%)	11 (45.8%)
ACOA	3 (42.9%)	4 (57.1%)
MCA	5 (71.4%)	2 (28.6%)
ACA	4 (57.1%)	3 (42.9%)
ICA	1 (33.3%)	2 (66.7%)
Posterior circulation	26 (49.1%)	27 (50.9%)
BA	9 (33.3%)	18 (67.7%)
VA	15 (62.5%)	9 (37.5%)
PICA	2 (100%)	0 (0)

### Clinical outcomes

The 37 fenestration patients with cerebral infarction were divided into two groups. Among them, 16 patients with cerebral infarctions in the blood supply area of the arterial fenestration comprised the fenestration-relevant cerebral infarctions group. The remaining 21 patients were assigned to the non-fenestration-relevant cerebral infarctions group. The risk factors of cerebral vascular fenestration patients with cerebral infarctions were compared between the two groups (Table 4). Fisher's precision probability test revealed that there were no differences in sex (68.8 vs. 76.2%, *P* = 0.716), history of stroke (25 vs. 23.8%, *P* = 1.000), hypertension (68.8 vs. 66.7%, *P* = 1.000), diabetes mellitus (37.5 vs. 28.6%, *P* = 0.726), dyslipidemia (12.5 vs. 9.5%, *P* = 1.000), atrial fibrillation (0 vs. 9.5%, *P* = 0.495), cerebral artery stenosis (87.5 vs. 76.2%, *P* = 0.674), hyperhomocysteinemia (25 vs. 9.5%, *P* = 0.371), coronary atherosclerotic heart disease (18.6 vs. 9.5%, *P* = 0.643), and recurrence of cerebral infarction (0 vs. 0, *P* = 1.000) between the two groups. Mann–Whitney U test did not detect any difference in age (*P* = 0.177) between the two groups. We also found that patients with an ischemic stroke focus more on the posterior circulation (87.5%) than on the anterior circulation (12.5%) in the fenestration-relevant cerebral infarction group. In addition, among the patients with fenestration in the anterior circulation fenestration, 54.2% of patients were <60 years old and 45.8% of patients were >60 years old, and among the patients with fenestration in the posterior circulation, 49.1% of patients were <60 years old and 50.9% of patients were >60 years old; hence, there seems to be no obvious age difference. After dual antiplatelet and lipid regulation therapy, no acute stroke or TIA occurred, while there were no adverse events emerged in 37 fenestration patients with cerebral infarctions during the follow-up.

**Table 4:** Differences in risk factors between fenestration-relevant and non-fenestration-relevant cerebral infarctions.

	**Total (*n =* 37)**	**Fenestration-relevant cerebral infarctions (*n =* 16)**	**Non-fenestration-relevant cerebral infarctions (*n =* 21)**	***p*-value**
Male, *n* (%)	27 (72.9%)	11 (68.8%)	16 (76.2%)	0.716^+^
Age, mean ± SD	56.76 ± 13.43	60.7 ± 12.1	53.8 ± 13.9	0.177^*^
Past history of stroke, *n* (%)	9 (24.3%)	4 (25%)	5 (23.8%)	1.00^+^
Hypertension, *n* (%)	25 (67.6%)	11 (68.8%)	14 (66.7%)	1.000^+^
Diabetes mellitus, *n* (%)	12 (32.4%)	6 (37.5%)	6 (28.6%)	0.726^+^
Dyslipidemia, *n* (%)	4 (10.8%)	2 (12.5%)	2 (9.5%)	1.000^+^
Atrial fibrillation, *n* (%)	2 (54.1)	0 (0)	2 (9.5%)	0.495^+^
Cerebral artery stenosis, *n* (%)	30 (81.1%)	14 (87.5%)	16 (76.2%)	0.674^+^
Hyperhomocysteinemia, *n* (%)	6 (16.2%)	4 (25%)	2 (9.5%)	0.371^+^
Coronary atherosclerotic heart disease, *n* (%)	5 (13.5%)	3 (18.6%)	2 (9.5%)	0.634^+^
Recurrence of cerebral infarction or TIA, *n* (%)	0 (0)	0 (0)	0 (0)	1.000^+^

Age is expressed by mean (± SD); n (%) represents the proportion of this part;

* is Mann–Whitney U test;

+ is Fisher's precision probability test; Statistical significance was considered at p < 0.05.

### Radiological characteristics

**Figure 1:**
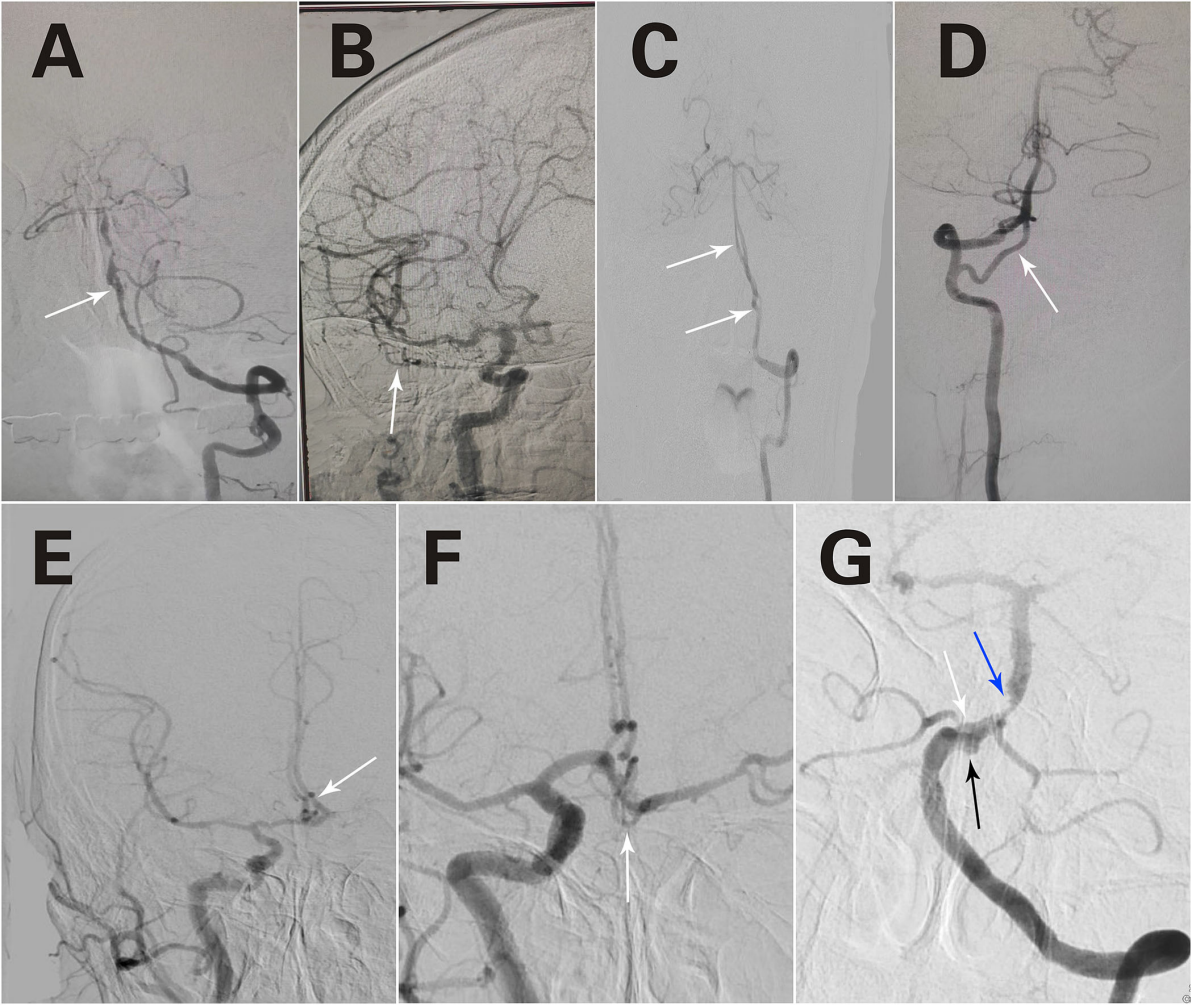
Different forms of fenestrations. A slit-like configuration **(A)**. A convex lens-like shape **(B)**. Duplication of arteries **(C)**. Irregular morphology **(D)**. Triple fenestrations of the anterior communicating artery **(E)**. An irregular morphology of the fenestration in the anterior communicating artery **(F)**. Two fenestrations are located in the VA and BA, respectively **(C)**. A fenestration was observed in BA, an aneurysm was located at the fenestration site, and basilar artery stenosis was observed **(G)**. The white arrows refer to fenestration; The black arrow refers to aneurysm; The blue arrow refers to artery stenosis.

**Figure 2:**
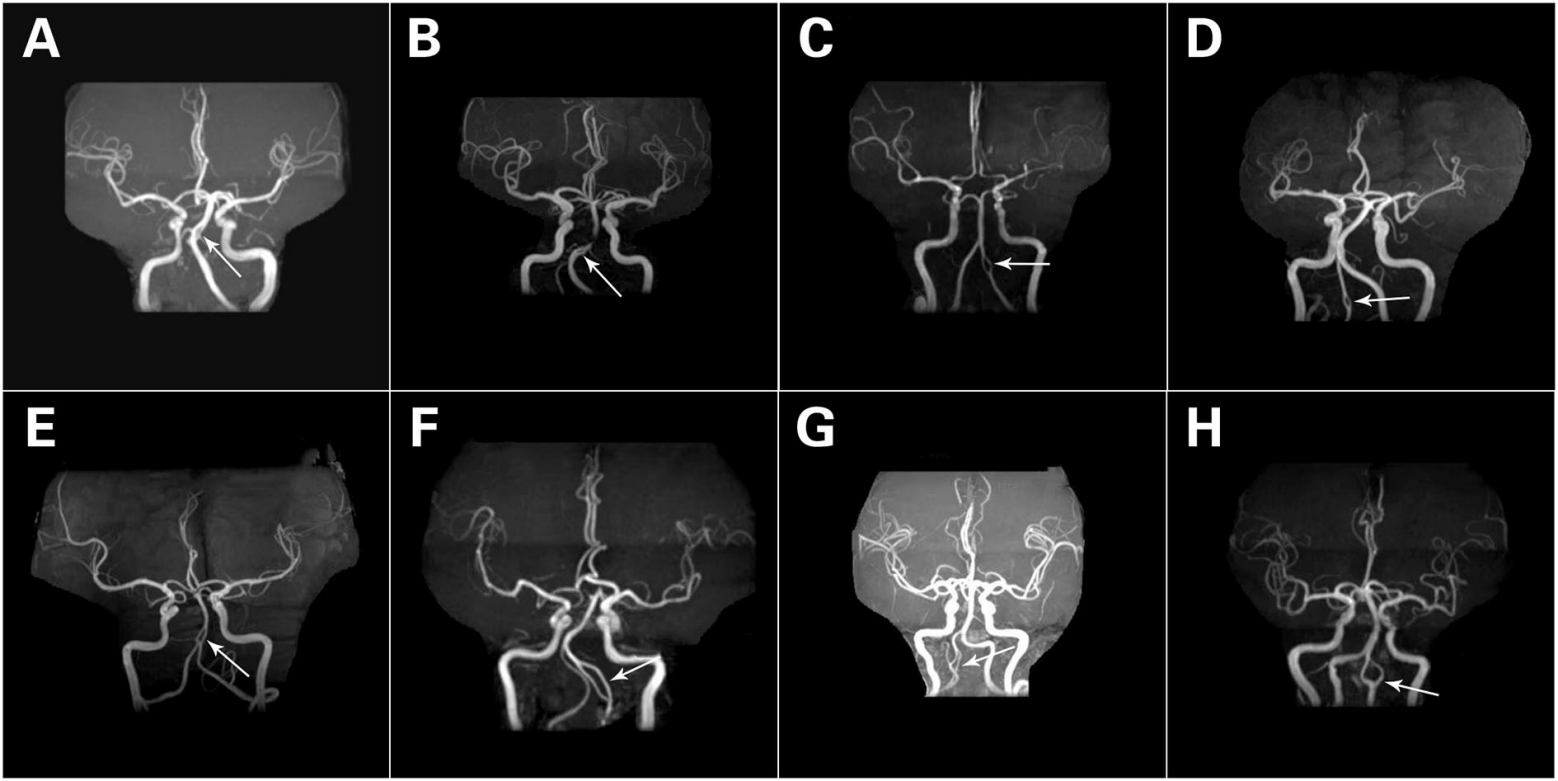
Different forms of fenestrations. A slit-like configuration **(A,B)**. A convex lens-like shape **(C,D)**. Duplication of arteries **(E,F)**. Irregular morphology **(G,H)**. The white arrows refer to fenestration.

A total of 80 fenestrations were detected in 76 patients. BA fenestrations accounted for 35% (28/80) of all fenestrations. The BA fenestrations were classified as follows: type I, 9 cases; type II, 9 cases; type III, 5 cases (of which one case had only one anterior inferior cerebellar artery and originated from the fenestration); and type IV, 5 cases. Moreover, among the other types of fenestrations, vertebral artery (VA) fenestration accounted for 30% (24/80), anterior communicating artery (ACOA) fenestration accounted for 11.25% (9/80), middle cerebral artery (MCA) fenestration accounted for 8.75% (7/80), anterior cerebral artery (ACA) fenestration accounted for 8.75% (7/80), internal carotid artery (ICA) fenestration accounted for 3.75% (3/80), and posterior inferior cerebellar artery (PICA) fenestration accounted for 2.5% (2/80) of all fenestrations. Among these fenestrations, 25 had a slit-like configuration (Figures 1A, 2A,B), while 29 had a convex lens-like shape (Figures 1B, 2C,D), 4 were duplications of arteries (Figures 1C, 2E,F), and 22 showed irregular morphology (Figures 1D, 2G,H). The types <5 mm accounted for 53.75% (43/80), 5–10 mm accounted for 15% (12/80), and ≥10 mm accounted for 31.25% (25/80) of fenestrations. The location, morphology, and size of the fenestrated vessels are shown in Table 5.

**Table 5:** Shape and differential location of cerebral artery fenestrations among the patients.

		**Location**							**Number (%)**
		**ACA**	**MCA**	**ACOA**	**VA**	**BA**	**PICA**	**ICA**	
Shape	Slit-like	1	1	2	5	15	0	1	25 (31.25%)
	Convex-lens-like	2	6	7	1	11	0	2	29 (36.25%)
	Duplication	4	0	0	0	0	0	0	4 (5%)
	Irregular	0	0	0	18	2	2	0	22 (27.5%)
Length	<5 mm	1	3	9	6	23	0	1	43 (53.75%)
	5–10 mm	4	4	0	0	2	0	2	12 (15%)
	≥10 mm	2	0	0	18	3	2	0	25 (31.25)
Number (%)		7 (8.75%)	7 (8.75%)	9 (11.25%)	24 (30%)	28 (35%)	2 (2.5%)	3 (3.75%)	80

ACA, anterior cerebral artery; MCA, middle cerebral artery; ACOA, anterior communicating artery; VA, vertebral artery; BA, basilar artery; ICA, internal carotid artery; PICA, posterior inferior cerebellar artery.

The results showed that most of these patients with fenestration are accompanied by other diseases: 37 patients had cerebral infarctions; 16 patients had cerebral infarctions in the blood supply area of the arterial fenestration; 10 patients had aneurysms, where only one aneurysm was detected at the site of fenestration; 4 patients had artery dissection, and 2 dissections were at the site of fenestrations; and 5 patients had moyamoya disease, and all “Smog-like” vessels were distal from fenestration.

In addition, special cases were detected among these patients. The first case presented triple fenestrations of the anterior communicating artery (Figure 1E). The second case had an irregular morphology of fenestration located in the anterior communicating artery (Figure 1F). The third case had bilateral fenestrations in the VA, accompanied by BA fenestration (Figure 1C). The fourth case was a BA fenestration, an aneurysm was located at the site of the fenestration, and basilar artery stenosis was observed simultaneously (Figure 1G).

## Discussion

The fenestration will cause hemodynamic changes in the vessels in which it is located, and whether it will cause the occurrence of other diseases is still unknown. Therefore, in the present study, we aimed to analyze the characteristics of cerebral artery fenestrations and explore the clinical relevance between cerebral vascular fenestration and cerebral infarction and aneurysm. The results showed that no clear clinical relevance was observed between fenestration and cerebral infarction, and no definite association was found between fenestration and aneurysm. Furthermore, we also found that fenestration in the posterior circulation may be related to cerebrovascular diseases.

Some studies have reported that cerebral artery fenestration is associated with cerebral ischemia or TIA (7, 10, 15–18), but the underlying pathogenesis is yet unclear. El Otmani et al. (15) showed that 75% of patients with ischemic stroke have pontine paramedian due to BA fenestration. Therefore, BA fenestration should be considered when patients without vascular risk factors (especially cerebral atherosclerosis) have pontine paramedian. Jeong et al. (10) reported 5 patients with MCA fenestration who had artery-related cerebral ischemia. The study proposed that the atherothrombotic process could be initiated because of the hemodynamic changes in the arterial branching site, progressing toward the proximal edge of the fenestration. The smaller limb of the fenestration is easily affected, which might be related to the low shear force and vortex of the vessel. In the current study, among the 76 fenestration patients, 37 patients had cerebral infarctions, and 16 patients had cerebral infarctions in the blood supply area of the arterial fenestration. This incidence of cerebral ischemia is higher than that in the general population (19), suggesting the involvement of fenestration in ischemic stroke. Considering that patients with more vascular risk factors have a higher risk of cerebral infarction. At the same time, if the patient has no risk factors and the cerebral infarction occurs in the blood supply area of the arterial fenestration, we considered that cerebral artery fenestration is associated with cerebral infarction. Therefore, we compared the risk factors between the fenestration-relevant and non-fenestration-relevant cerebral infarctions groups and concluded that there were no differences in risk factors between the two groups. Based on the above findings, we found that there is no clear clinical relevance between fenestration and cerebral infarction. Then, we analyzed the vascular stenosis and the location of fenestration in these 16 patients and identified 2 patients with fenestration and vascular stenosis in the same vessel, meanwhile, the two patients had no risk factors. For the relationship between cerebral artery fenestration and vascular stenosis, we made the following guesses: cerebral artery fenestrations increase the bending of vessels, making the angle of the bend of the vessels become sharp, and causing hemodynamic changes, followed by damaged arterial intima and also forming atherosclerotic plaque, and this may further cause intracranial artery stenosis. Furthermore, for those 37 fenestration patients with cerebral infarctions who received the treatment of dual antiplatelet and lipid regulation, no acute stroke or TIA occurred, and no adverse events emerged during the follow-up. This phenomenon suggested that long-term antiplatelet and statin therapy may be safe and effective for cerebral infarctions patients with fenestrations.

Previous studies have described the connection between the fenestration of the intracranial artery and cerebral aneurysm (20–23), while some suggested that altered flow dynamics in the presence of fenestration may promote aneurysm development, although the exact relevance is not well-defined. In the present study, 10 aneurysms were detected in 9 (11.8%) patients. This rate is low compared with the study by Cooke et al. (1), in which 60.5% of the patients with fenestrations had at least one aneurysm, and compared with van Rooij et al. (23), it was 31%. Notably, the incidence of aneurysms observed in the present and previous studies (15–60%) (1, 4, 5, 23, 24) is much higher than that in the general population (1–2%) (25), suggesting an association between fenestration and aneurysm. But in our study, only one aneurysm was at the site of fenestration, and the remaining 9 aneurysms were different from the fenestrations, indicating that there is no clinical correlation between fenestration and aneurysm. This is consistent with the research results of Cooke et al. (1) and Krystkiewicz et al. (21). Nonetheless, in 8 patients, the length of the fenestrated vessel branch was <5 mm, and in 1 patient, the length of the fenestrated vessel branch was >10 mm. Typically, the size of the fenestrations was small in these 8 patients, which might cause slight hemodynamic changes that are not sufficient for aneurysm formation, necessitating further exploration. Furthermore, this was a retrospective study of already diagnosed cases, and hemodynamic changes in these patients were not detected. Also, accurate measurements of hemodynamics are not available.

In addition, 4 patients with cerebral artery dissection were found. Among them, 2 dissections were located at the site of fenestrations, and 2 dissections were different from fenestration. The clinical relevance between cerebral artery dissection and fenestration has not been reported previously. Although fenestration alters vascular hemodynamics, whether this will lead to dissection still needs to be investigated further.

In our study, we also found that the ischemia focus of the 16 patients was located in the territories of fenestrated arteries, including 12.5% (2/16) in the anterior circulation and 87.5% (14/16) in the posterior circulation, which is similar to the finding of Ye et al. (26), indicating that vertebrobasilar fenestrations may be related to cerebrovascular diseases. In addition, considering that the incidence of cerebral infarction is higher in people aged over 60 years. Therefore, we compared the incidence of fenestration in anterior or posterior circulation between the two groups of patients older than 60 years and younger than 60 years. We found no difference in age in fenestration. This finding will help us to further analyze the relationship between fenestration and cerebral infarction.

Nevertheless, the present study has some limitations. First, it was a retrospective study with a small sample size. Second, most patients had different degrees of cerebrovascular stenosis, which increases the incidence of cerebral infarction. Finally, since the radiologists were not explicitly looking for fenestrations when they read the images, it is possible that many patients were not included in this study. Hence, a multicenter study with a large number of patients would provide definite data about the location, morphology, and configuration of cerebral artery fenestrations.

## Conclusion

In the current study, basilar artery fenestration is the most common form of the condition. Fenestration can be divided into four variants, namely, slit-like shape, convex lens-like shape, duplicated artery, and other irregular shape fenestrations. The most common types were slit- and convex lens-like shapes. In this retrospective study, we did not observe a clear clinical relevance between fenestration and cerebral infarction. Also, we did not find a definite association between fenestration and aneurysm. Interestingly, fenestration in the posterior circulation may be related to cerebrovascular diseases. Furthermore, for those fenestration patients with cerebral infarctions, the long-term antiplatelet and lipid-lowering treatment may be safe and effective. Finally, a large multicenter study could be used to explore the clinical relevance between cerebral artery fenestration and other associated vascular diseases.

## Data availability statement

The original contributions presented in the study are included in the article/supplementary material, further inquiries can be directed to the corresponding author/s.

## Ethics statement

The studies involving human participants were reviewed and approved by the Institutional Review Board of Yantai Yuhuangding Hospital. The patients/participants provided their written informed consent to participate in this study. Written informed consent was obtained from the individual(s) for the publication of any potentially identifiable images or data included in this article.

## Author contributions

ZL, LX, and XC contributed to the study concept and design, data analysis, interpretation, and also guarantors of this work and have full access to the data. LX and XC managed the literature search and analysis. LX undertook the statistical analysis and wrote the first draft of the manuscript. HW, XC, and WX collected and analyzed the data. All authors contributed to the article and approved the submitted version.
